# Characteristic bimodal profiles of RNA polymerase II at thousands of active mammalian promoters

**DOI:** 10.1186/gb-2014-15-6-r85

**Published:** 2014-06-12

**Authors:** Mathieu Quinodoz, Cédric Gobet, Felix Naef, Kyle B Gustafson

**Affiliations:** 1 The Institute of Bioengineering, School of Life Sciences, École Polytechnique Fédérale de Lausanne (EPFL), 1015 Lausanne, Switzerland; 2 Center for Integrated Genomics, University of Lausanne, Génopode, 1015 Lausanne, Switzerland

## Abstract

**Background:**

In mammals, ChIP-seq studies of RNA polymerase II (PolII) occupancy have been performed to reveal how recruitment, initiation and pausing of PolII may control transcription rates, but the focus is rarely on obtaining finely resolved profiles that can portray the progression of PolII through sequential promoter states.

**Results:**

Here, we analyze PolII binding profiles from high-coverage ChIP-seq on promoters of actively transcribed genes in mouse and humans. We show that the enrichment of PolII near transcription start sites exhibits a stereotypical bimodal structure, with one peak near active transcription start sites and a second peak 110 base pairs downstream from the first. Using an empirical model that reliably quantifies the spatial PolII signal, gene by gene, we show that the first PolII peak allows for refined positioning of transcription start sites, which is corroborated by mRNA sequencing. This bimodal signature is found both in mouse and humans. Analysis of the pausing-related factors NELF and DSIF suggests that the downstream peak reflects widespread pausing at the +1 nucleosome barrier. Several features of the bimodal pattern are correlated with sequence features such as CpG content and TATA boxes, as well as the histone mark H3K4me3.

**Conclusions:**

We thus show how high coverage DNA sequencing experiments can reveal as-yet unnoticed bimodal spatial features of PolII accumulation that are frequent at individual mammalian genes and reminiscent of transcription initiation and pausing. The initiation-pausing hypothesis is corroborated by evidence from run-on sequencing and immunoprecipitation in other cell types and species.

## Background

Regulation of mRNA transcription is dependent on the recruitment and engagement of available RNA polymerase II (PolII) to the appropriate genes at the required times. A coordinated series of events involving PolII in complex with both general and specific transcription factors is known to start with recruitment [1,2], continue through promoter opening [3], then promoter escape, pausing [4,5], and, finally, release into productive elongation [6]. A variety of techniques are informative for localizing PolII and thus inferring the details of transcription regulation at these checkpoints.

Previous results show that pausing before productive elongation is a general feature of transcription regulation in eukaryotes. However, since the promoter sequences and nucleosome organization are distinct between different species [7], the details of regulation should be distinctly considered. Originally in *Drosophila melanogaster*, localization of a PolII peak at 40±20 base pair (bp) downstream from the *hsp70* transcription start site (TSS) was inferred from the length of terminated run-on transcripts [8]. This 40-bp figure for *Drosophila* is supported by genome-wide assays in at least two categories: run-on sequencing and chromatin immunoprecipitation (ChIP). Run-on sequencing of nascent RNA as a proxy for the location of PolII showed a peak at 50 bp downstream of the TSS in *Drosophila*[9]. This 50-bp number is generally supported in mammalian cells [10,11]. On the other hand, ChIP with deep sequencing (ChIP-seq) generally showed that PolII occupies a 200-bp-wide peak centered near 50 bp downstream of the TSS at most active promoters in both mammalian cells and *Drosophila*[12-18].

At near base-pair resolution, a lambda exonuclease digestion of immunoprecipitated chromatin (ChIP-exo) was used [19] to visualize pre-initiation complexes (PICs) and PolII in *Saccharomyces cerevisiae*, concluding that PICs are centered at 30 to 40 bp downstream of TATA boxes. Yet another technique, permanganate-ChIP-seq, has also been used as a signal for open PICs in *Drosophila*[20] to infer pausing at +50 bp. Notably, the permanganate peak in *Drosophila* seems to be 50 bp downstream from the TSS but roughly 50 bp upstream from the PolII peak, which is slightly upstream from the first downstream nucleosome at 135 bp [7]. In mammalian cells, a combination [21] of strand-specific RNA deep sequencing (RNA-seq), micrococcal nuclease digestion with sequencing (MNase-seq) and PolII ChIP-seq shows a 150-bp-wide PolII ChIP-seq peak centered near 50 bp, upstream of the +1 nucleosome. Generally, it seems that nuclear run-on assays give a sharp pausing peak at 50 bp, while ChIP-seq assays give broad peaks centered near 50 bp.

Here, we characterize the occupancy of PolII at known TSSs on the mammalian genome with a higher spatial resolution due to a next-generation sequencing machine and careful alignment of the mapped reads. At promoters, the improvement in resolution of PolII occupancy identifies a bimodal pattern, centered at 50 bp downstream from the consensus TSS but with two distinct peaks genome-wide. We reason that these peaks are very likely to represent initiated and paused polymerase as they are consistent with published results but are visible only with higher resolution. We eliminate several alternative explanations using our murine liver dataset and support our argument with a reanalysis of published PolII, negative elongation factor (NELF) and DRB-sensitivity inducing factor (DSIF) ChIP-seq in HeLa cells. This evidence for separate initiation and pausing prompted us to create a novel quantification of both the rate of PolII promoter escape into the paused state as well as the rate of pausing release into elongation. We also numerically model features of the bimodal promoter-proximal PolII profile and compare with an RNA-seq dataset. Finally, we systematically report quantitative relationships between DNA sequence, PolII, H3K4me3 and mRNA expression levels.

## Results and discussion

### PolII shows a generic bimodal density at transcription start sites

**Figure 1:**
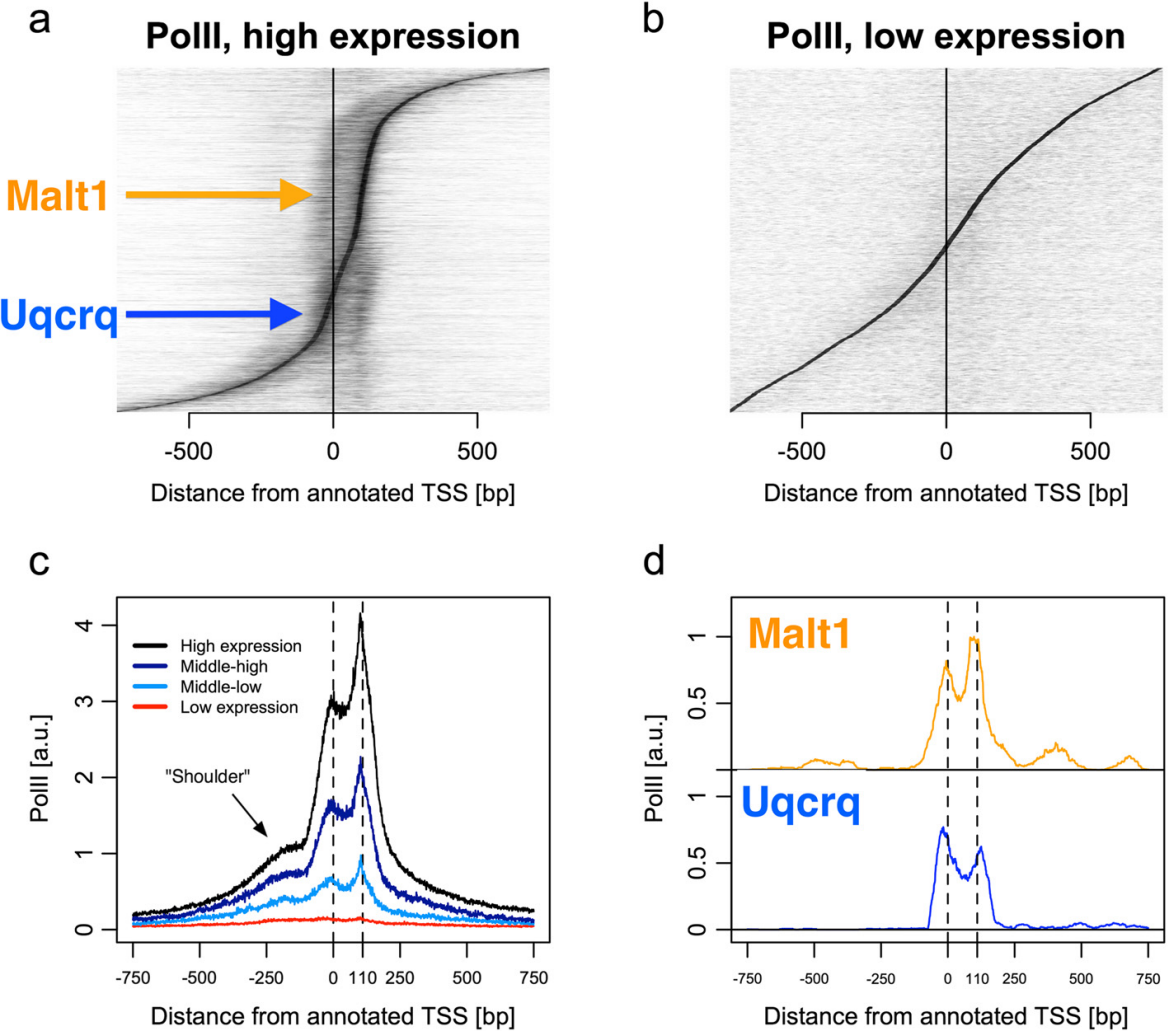
**High-resolution PolII profiles show distinct accumulation peaks at the TSS and + 110 ± 20 bp downstream.** Sorted ChIP-seq profiles and genome-averaged profiles of normalized PolII signal on promoters are displayed from 5^′^ to 3^′^ centered at each TSS. Transcripts are separated by microarray expression into lowly expressed (<6.0 microarray units), moderately expressed (between 6.0 and 8.0 units) and highly expressed (>8.0 units) (see also Figure 2b). **(a, b)** Stacked profiles, sorted vertically by position of maximum signal, with signal strength normalized to give the same shade of grey at the maximum for each profile. **(c)** Genome-averaged profile of PolII occupancy for highly expressed (black), moderately expressed divided into two quantiles (two shades of blue) and lowly expressed (red) transcripts. The two dashed lines indicate the two main peaks at TSS and TSS + 110. **(d)** Examples of genes with different peak-height ratios, not normalized to their maxima: Malt1 (orange, peak-height ratio 1.28) and Uqcrq (blue, peak-height ratio 0.93) as indicated in **(a)**. a.u., arbitrary units.

We obtained high-coverage ChIP-seq profiles with five times more sequencing coverage than previously published for a library of murine hepatic chromatin [22]. We examined all transcripts and noticed a group of about 10,000 expressed transcripts (determined from microarray probes, see Materials and methods). The PolII profiles for highly expressed transcripts show a characteristic bimodal, or double-peaked, pattern (Figure 1a,c) previously unobserved to our knowledge. The prominence of the profile gradually attenuates with decreasing microarray expression (Figure 1b,c). We saw the same pattern for PolII ChIP-seq from each of seven similar ChIP-seq libraries that we sequenced with higher coverage than previously done [22]. We now focus on this unique observation of bimodal PolII occupancy in the region just downstream of the TSS. All gene selections for each figure are specified in Additional file 1.

**Figure 2:**
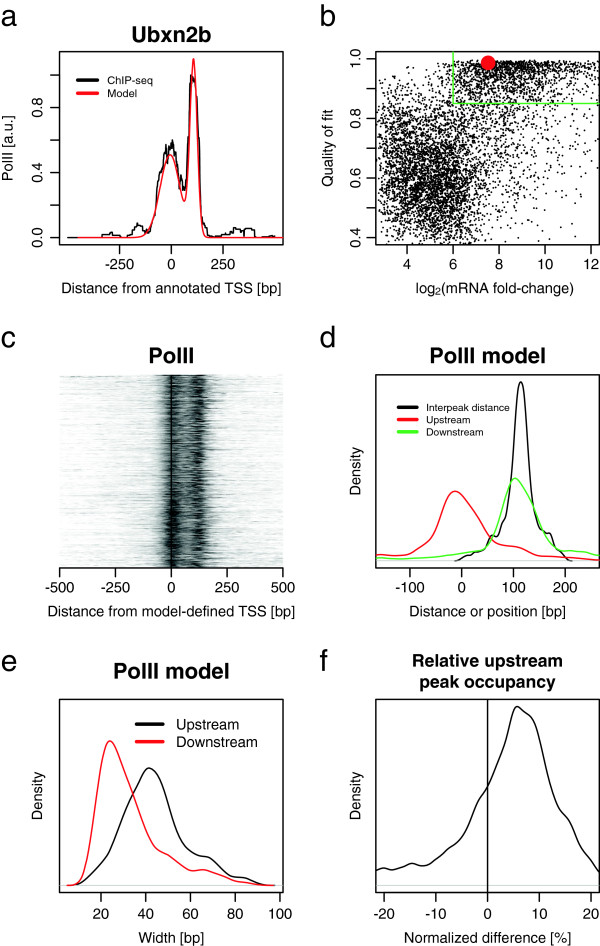
**Promoter-proximal PolII profiles of expressed transcripts can be modeled gene-by-gene.** Quantification of PolII bimodal peaks in individual promoters. **(a)** An example of fitting a linear combination of two Gaussian profiles to the PolII ChIP-seq signal near the TSS of Ubxn2b. The parameters for this example are: inter-peak distance 113 bp, upstream peak width 48 bp, downstream peak width 18 bp, peak-height ratio 2.16, difference in occupancy + 28% for second peak, shift from annotated TSS 2 bp and quality of fit 0.99. **(b)** Quality of the model fit correlates well with the mRNA microarray signal. Ubxn2b is highlighted with a red dot (6,475 isolated genes). **(c)** Overhead view of normalized profiles of PolII at TSS after re-centering all promoters according to the TSS predicted by the model (1,475 expressed genes with quality score >0.85). **(d)** Distribution of the inter-peak distances and peak positions (same genes as **(c)**). **(e)** Distribution of the fitted width (standard deviation) of profile features (same genes as **(c)**). **(f)** Distribution of the difference in occupancy in upstream peak defined as (area under the upstream peak minus area under the downstream peak)/max(area under the upstream peak, area under the downstream peak) (same genes as **(c)**). a.u., arbitrary units.

The upstream peak in this bimodal pattern is centered near TSSs. Downstream, a second peak typically at +110±20 bp is consistently observed. This 20-bp uncertainty mainly reflects variability in the position of the upstream peak (see Figures 1a and 2d,e). The position and shape of the bimodal pattern is a refinement to observations of PolII promoter-proximal profiles in other reports [3,5,10,11,14-16,21]. There are two reasons for this improvement. The first is the fivefold increase in the number of mapped reads on the HiSeq Illumina sequencer. Second, the reads were carefully aligned according to the insert size and the read length (see Materials and methods and Additional file 2).

Individual high-coverage PolII profiles (Figure 1d, Additional file 3) clearly show that the bimodal promoter-proximal profile does not reflect population heterogeneity. On the contrary, many genes individually exhibit both peaks separated by a consistent distance. We denote the occupancy of the upstream peak as *p*_*u*_ (total number of reads) and the downstream peak occupancy as *p*_*d*_. The normalized occupancy difference is defined as *ρ*≡(*p*_*u*_-*p*_*d*_)/ max(*p*_*u*_,*p*_*d*_). Of the set of highly expressed genes in Figure 1a, approximately 30*%* have *ρ*<0 (an example in Figure 1d is Uqcrq) and approximately 70*%* have *ρ*>0 (an example in Figure 1d is Malt1). The position of the profile relative to the TSS tends to vary slightly, as indicated by the S-shape in Figure 1a, in which genes are vertically sorted according to the position of maximal accumulation.

**Figure 3:**
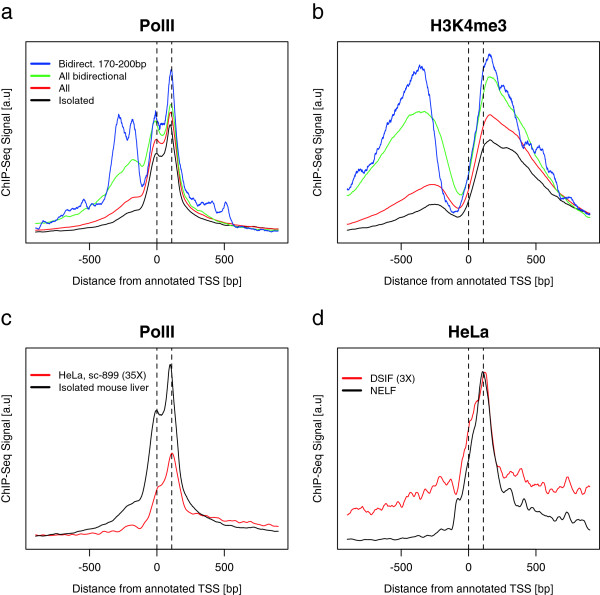
**ChIP-seq signal near TSS for mouse liver and HeLa cells. (a, b)** ChIP-seq profiles near TSS (mm9 genome) for highly expressed transcripts in mouse liver, separated into the following categories: all expressed (red, 10,111 genes, >6.0 microarray units), expressed and isolated with no other TSS or PAS within 1 kb (black, 3,070 genes), those expressed with an oppositely directed TSS less than 1 kb upstream (green, 1,605 genes) and those expressed with an oppositely directed TSS between 170 and 200 bp upstream (blue, 91 genes). **(a)** PolII averaged signal, arbitrary units. **(b)** H3K4me3 averaged signal, arbitrary units. **(c, d)** The promoter-proximal bimodal profile with separation of 110 bp is apparent also for HeLa cells [23]. Vertical lines are given at the consensus TSS (hg19 genome) and at TSS + 110 bp. **(c)** Profiles of PolII in mouse liver (black) and HeLa cells using a different antibody (sc-899 instead of our sc-67318) (red). Transcripts are chosen to be isolated from nearby transcripts and to have non-zero RNA-seq signal (see Materials and methods). HeLa coverage is multiplied by 35 to be on the same scale as the higher coverage mouse liver data. **(d)** In HeLa cells, both pausing factors NELF and DSIF co-localize with PolII as expected, with a peak at TSS + 110 bp. DSIF coverage is multiplied by 3 to be on the same scale as NELF. a.u., arbitrary units.

In support of this result, we have also reanalyzed a published ChIP-seq dataset for PolII in a HeLa cell line [23], which clearly shows a very similar bimodal pattern (peak at the TSS and taller peak at TSS +110 bp) for PolII in a genome-wide profile for active genes (Figure 3c). Sequencing coverage is not high enough to observe whether or not a bimodal pattern exists for individual genes in the HeLa dataset. However, we also see a strong peak at TSS +110 bp in the profiles of pausing factors DSIF and NELF, which are known to associate with PolII [23,24] (Figure 3d). The interpretation of the downstream peak as a pausing peak is supported by the unimodal shape of the pausing factor occupancies. Thus our result for the mouse seems to be more generally valid for mammals and is not strictly dependent on the choice of PolII antibody or cell type.

Two fundamental assumptions should be considered. First, the epitope for the RPB2 antibodies are assumed to be similarly accessible in the initiated and paused state. Second, given the large population of cells processed, the two peaks may represent either multiple polymerases on the same region of DNA or multiple occupancy states of the same gene across many cells. Despite these ambiguities, it is clear that this consistent profile in individual promoters represents a pattern of occupancy for PolII in the region known to be associated with initiation and pausing. We present evidence that some explanations for this feature are unlikely to be sufficient. First we rule out interference in the signal due to nearby transcriptional activity in the opposite direction.

### Bimodal PolII occupancy near the transcription start site persists after filtering neighboring transcripts

A bimodal pattern in PolII ChIP-seq occupancy was also recently revealed by ChIP-seq of PolII for mammalian cells [21]. In that study, the second peak at 200 to 300 bp upstream of the TSS was attributed to nearby, oppositely directed transcription [25]. We identified this phenomenon with the ‘shoulder’ feature 200 bp upstream of the TSS in our profile (Figure 1c). We removed this shoulder by isolating transcripts with the following properties: nearby promoters, a neighboring polyadenylation site (PAS), or an alternative TSS from the (NCBI37) database of transcripts (see Materials and methods). When these are removed from the genome-wide profile, only the shoulder is diminished (Figure 3a), while the bimodal pattern is retained. In fact, when we inversely restrict the set of transcripts to include only bidirectional promoters within 170 to 200 bp of the TSS, the bimodal PolII pattern is clearly reflected in the opposite direction (Figure 3a, blue curve). The forward and reverse bimodal profiles are distinct: one starting at the consensus TSS and one in the opposite direction at -200 bp. This is expected because the oppositely directed TSSs are selected near -200 bp.

Obvious differences in ChIP-seq profiles due to the presence of bidirectional promoters can also be seen in H3K4me3 (Figure 3b), a known promoter-proximal mark for active transcription [3,22,26,27]. Profiles of H3K4me3 near the TSS are sharply peaked at both +150 and -400 bp relative to the reference TSS. The position of the downstream peak maximum does not change significantly when bidirectional promoter signals are removed, but the upstream peak decreases in amplitude and shifts downstream. RNA-seq profiles (see Materials and methods) show a similar symmetrical upstream signal that vanishes when bidirectional promoters are removed (Additional file 4a).

These considerations, from PolII, H3K4me3 and RNA-seq, convince us that bidirectional promoters are responsible for the majority of the shoulder signal (Figure 1c) in the genomic PolII profile at TSS - 200 bp [10,14]. Indeed, the genome-wide distribution of nearest opposite promoters shows a peak at TSS - 200 bp (Additional file 4b). Upon elimination of bidirectional promoters within less than 1,000 bp of each TSS, the bimodal PolII profile at the TSS remains. Therefore, isolated TSSs, without signals from nearby genes, are used for all following analyses. The remaining signal upstream from the TSS following this filter (Figure 3a, black curve) may be due to either unannotated or unidentified bidirectional promoters and divergent transcription.

### Quantitative modeling of PolII pattern reveals conserved features

To describe and explain the conserved features in the bimodal profiles, we modeled them with a sum of two Gaussian distributions (see Materials and methods). In our case, a small number of parameters can be used to describe the Gaussian model: the inter-peak distance, the ratio of peak heights, the peak widths and the distance from the annotated TSS to the upstream peak. Moreover, by assigning a quality score between 0 and 1 to the accuracy of the fit, we can sort transcripts according to how well their promoter-proximal PolII occupancy matches the Gaussian model. A single PolII ChIP-seq profile and its model fit are shown in Figure 2a for UBX domain protein 2B (Ubxn2b), a particularly high-quality example. Generally, the computed quality of fit and mRNA expression show a separation into two populations (Figure 2b, red dot for Ubxn2b). About 65% of expressed transcripts are in the better-quality population.

We find the inter-peak distance is more conserved than the distance between the upstream peak and the annotated TSS. The bimodal pattern displays a remarkably consistent shape (Figure 2c) when PolII profiles from the well-fitted expressed population (green box in Figure 2b) are shifted, using the model fit, to align the downstream peaks. The inter-peak distance is sharply peaked at +110±20 bp (Figure 2d, black curve). The upstream and downstream peaks are centered near the TSSs and at 110 bp (Figure 2d, red and green curves). The upstream peak tends to be about twice as wide as the downstream peak (Figure 2e). The distribution of the normalized occupancy difference shows a maximum at *ρ*_max_≃6*%*, reflecting a moderately higher occupancy of PolII at the downstream peak on average across the genome (Figure 2f).

Since expressed transcripts are well-correlated with a clearly distinguished downstream peak (Figure 1) and this peak is consistently nearby but distinct from the TSS, it seems to signify paused polymerase. The upstream and downstream enrichments, together with the density of tags in the gene body, can define two pausing indices. For example, the promoter-escape index is identified with the normalized occupancy difference *ρ*. A putative pause-release index, *π*, could be the ratio of the gene body tag density to the downstream peak tag density, similar to the previously-defined escape index [28] (also see [10,13]). When computing *ρ*, to avoid false-paused genes, we note that special care must be taken to avoid bidirectional genes and genes with a nearby TSS, as we described above.

### RNA-seq corroborates shifting of transcription start sites

We find that the location of the TSS indicated by the upstream half of the bimodal PolII signal sometimes disagrees by 50 to 200 bp with annotated TSSs. We showed (Figure 2d) how the inter-peak *spacing* in the PolII promoter profile is more sharply conserved than the *position* of the upstream peak near the TSS. This demonstrates the uncertainty inherent in the position of the TSS [29,30]. We thus used non-strand-specific RNA deep-sequencing (see Materials and methods) from the same experimental conditions as the ChIP-seq data to attempt a re-estimation of each TSS.

**Figure 4:**
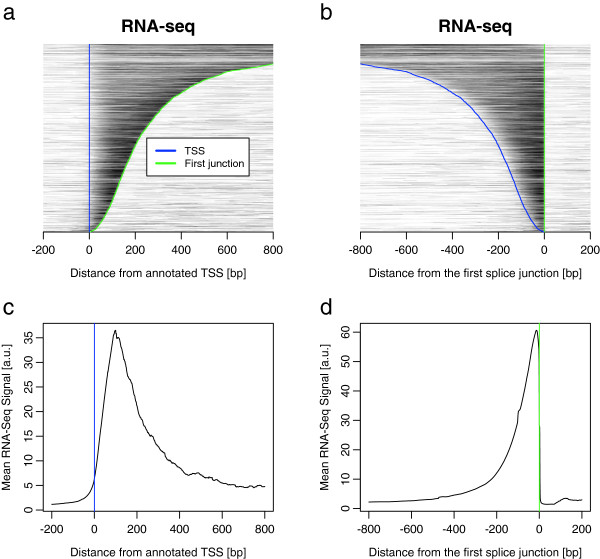
**RNA-seq demonstrates precision of TSS relative to first splice junction. (a, b)** Overhead view of individual profiles of RNA-seq signal normalized to self-maximum, grey-shaded as in Figure 1 (paired-end read coverage pileup, STAR mapping) sorted vertically by the length of the first exon. **(a)** Signal aligned by TSS and **(b)** aligned by first splice junctions (2,790 expressed and isolated genes and without the top 10% highest signal genes). **(c, d)** Averaged profiles of RNA-seq, **(c)** aligned by TSS and **(d)** aligned by first junction. These are simply the mean profiles computed from the set of profiles in **(a)** and **(b)**. a.u., arbitrary units.

**Figure 5:**
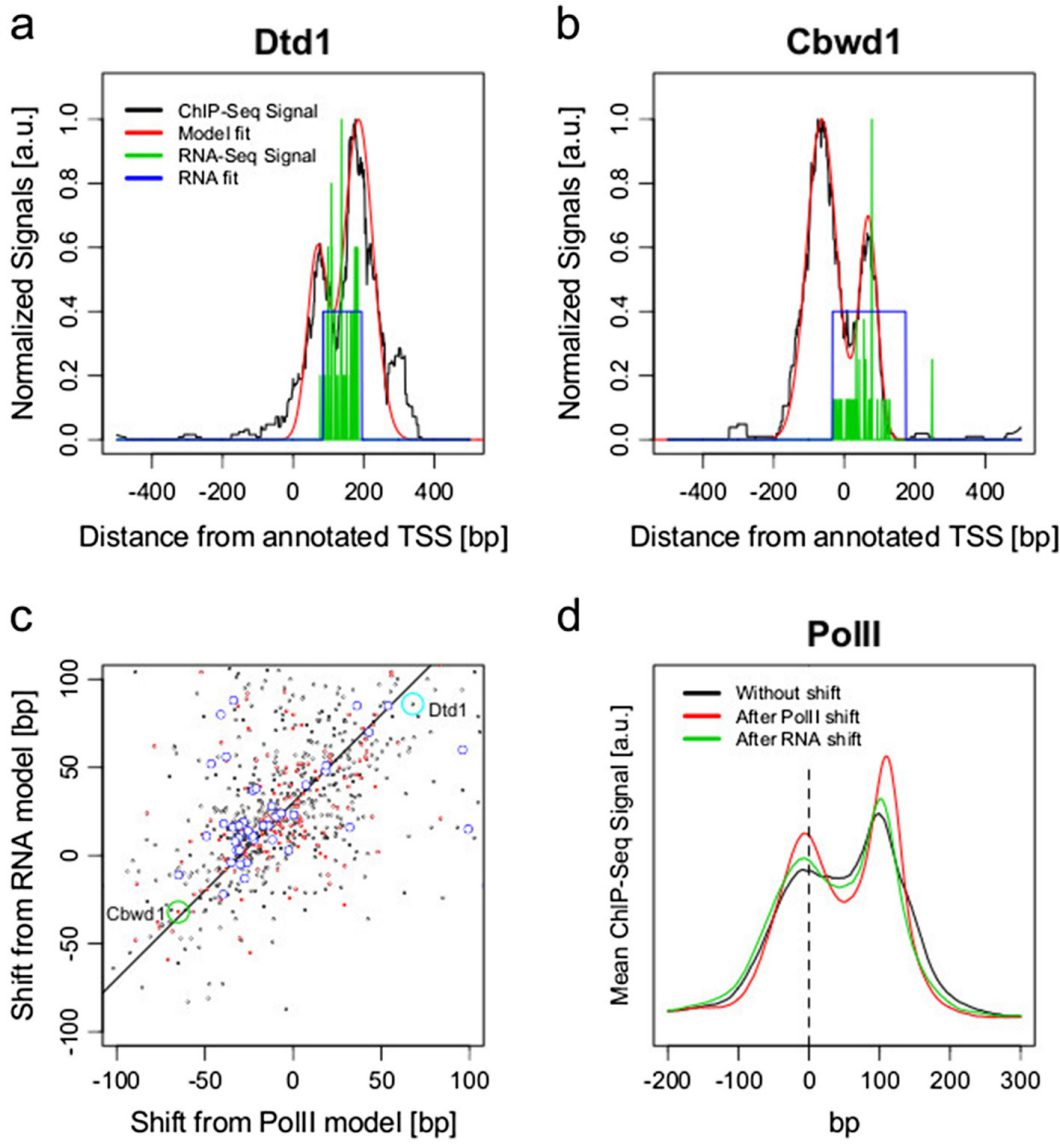
**PolII and RNA-seq model fits are correlated. (a, b)** Examples of two imprecise TSSs for **(a)** Dtd1 and **(b)** Cbwd1, showing the sum of Gaussians model fit (red) to PolII signal and rectangular function model fit (blue) for the RNA signal. **(c)** Correlation between the shifts defined by PolII and RNA-seq models for strict (black: 731 genes with *R*^2^=0.56, *P*<2.2×10^-16^) and very strict (red: 183 genes with *R*^2^=0.72, *P*<2.2×10^-16^) values of the fitting-quality criterion and TATA genes (blue). Dtd1 and Cbwd1 are highlighted with a light blue and a green circle, respectively. **(d)** Averaged profiles of PolII occupancy near TSS with no shift, RNA-seq model shift or PolII model shift to align first peak positions (731 genes). a.u., arbitrary units.

RNA-seq coverage is shown in Figure 4 near the annotated TSS and first splice junction. The first splice junction is used as an example of a naturally sharply-defined feature. We observe a gradual onset of mRNA reads after the TSS (Figure 4a,c) compared to a sharp transition when mRNA coverage is aligned at the first splice junction (Figure 4b,d). This difference may be explained by multiple factors: the tissue-dependency of annotated databases of TSSs [29], dispersion of initiation events [30,31] and other inconsistencies in published annotations for some genes. This signal may be affected by the details of fragmentation and primer design in the RNA-seq protocol, though the small errors due to these effects do not change our conclusion (see below and [32,33]).The TSS may be inferred by fitting the RNA-seq profile with a rectangular model function (example in Figure 5a,b, blue step) (see Materials and methods). The examples of Dtd1 and Cbwd1 in Figure 5a,b show the realignment of the TSS using this method. Moreover, the TSS computed from the position of the upstream peak in the PolII data is often similar to the TSS from the RNA-seq model (Figure 5c), as indicated by the clear linear correlation between modeled TSS shifts. We note that promoters with strong TATA boxes (TATA score >-5) generally have smaller inferred TSS shifts (Figure 5c, 25% smaller standard deviation for blue dots).

There is, however, a systematic bias (approximately 30 bp) between the models of the TSS obtained by RNA-seq (Figure 5c, diagonal line) compared to the PolII ChIP-seq. Half of this bias is due to the genome-averaged PolII peak at the TSSs being on average 15 bp upstream of the consensus TSS (Figure 2d). The other half is due to the RNA-seq read coverage starting on average 15 bp downstream of the consensus TSS, possibly due to bias against sequencing small fragments from the ends of mRNA or error due to random hexamer priming [32,33]. The TSS estimate from RNA-seq and PolII are correlated (*R*^2^=0.56, *P*<2.2×10^-16^), considering this systematic error. We also observed a correlation (*R*^2^=0.41, *P*<2.2×10^-16^) between PolII TSS accuracy and CAGE [34,35] transcription initiation sites (Additional file 5). The CAGE peak is tightly centered on the annotated TSS, while the first peak from the PolII model is 15 bp upstream, as already noted (Figure 2d). Finally, as an indication of the genome-wide effect of re-estimating the TSS, we note that the averaged profile of PolII becomes more sharply peaked when the modeled shifts are applied (Figure 5d).

### Transcription start site PolII signal is correlated with DNA sequence and RNA expression

We now consider further the link between mRNA microarray expression and DNA sequence elements known to be related to transcription regulation [36], such as CpG islands [37] and TATA boxes [38]. This connection was also recently explored in a meta-analysis of data from human cell lines for many chromatin marks [39].

**Figure 6:**
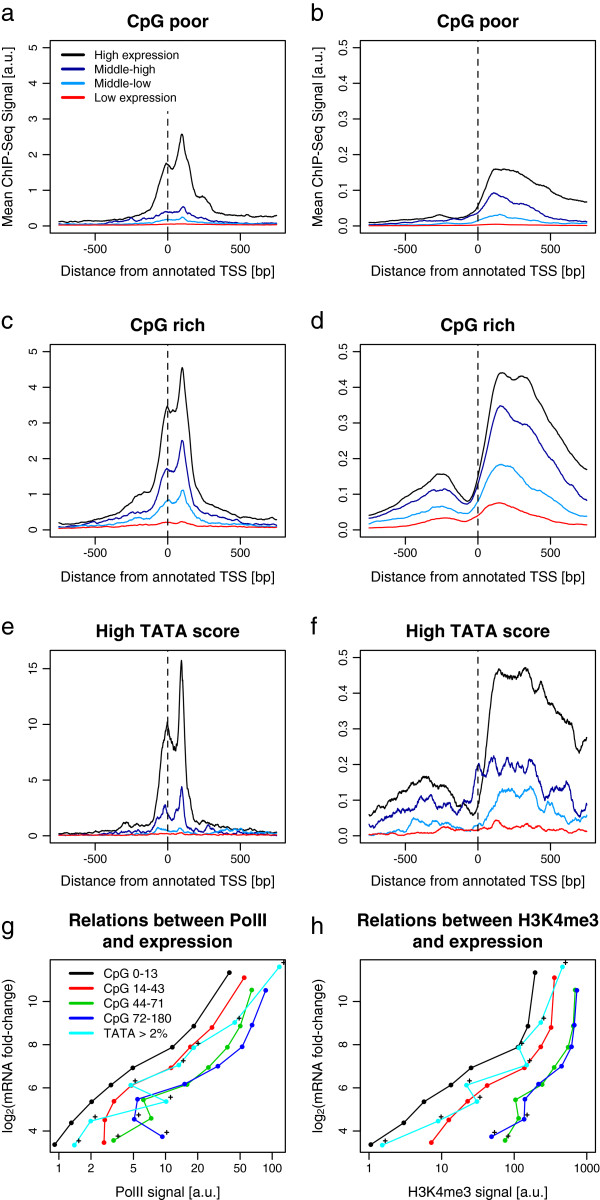
**PolII and H3K4me3 occupancy at TSSs correlate with CpG number, TATA score and mRNA expression. (a–f)** Genomic profiles of ChIP-seq around TSSs for PolII **(a, c, e)** and H3K4me3 **(b, d, f)**. Expression thresholds on microarray data are 6.0 and 8.0 as in Figure 1. **(a, b)** CpG-poor promoters have less than 50 CpG counts in TSS ± 1 kb (3,873 genes). **(c, d)** CpG-rich promoters have more than 50 CpG counts (2,520 genes). **(f)** High TATA score promoters have the top 2% of TATA scores (98 genes). **(g, h)** Microarray signal as a function of PolII ChIP-seq signals **(g)** and H3K4me3 ChIP-seq signals **(h)** within 1 kb of a TSS stratified by the CpG content of promoter sequences (see Materials and methods). A cross indicates that the point represents less than 1% of all TSSs. a.u., arbitrary units.

The bimodal pattern for PolII has a significantly higher amplitude for higher levels of mRNA expression (Figure 6a,c,e) regardless of the presence of CpG islands or TATA boxes. However, the peak at the TSS is much sharper for TATA genes, confirming a more sensitive response for PolII recruitment at TATA boxes compared to more dispersed CpG-rich regions (i.e. [29]). Indeed, the TATA genes are less often expressed (30% of high TATA score promoters are highly expressed, compared to 50% overall) but when they are expressed, they tend to be more highly expressed (top 5% TATA genes are 2.7 times more expressed than top 5% non-TATA genes). The H3K4me3 pattern is also significantly more pronounced for higher mRNA expression (Figure 6b,d,f). There is a larger H3K4me3 signal, both upstream and downstream of the TSS, for CpG-rich genes compared to CpG-poor genes, even for lowly expressed genes.

We also examined the relationship between PolII, H3K4me3 and mRNA microarray expression (Figure 6g,h). The dependence of mRNA expression on PolII or H3K4me3 signal within 1 kb of the TSS is logarithmic for low levels of expression. There is a loss of predictive capacity for higher expression, in the sense that multiple expression levels correspond to the same occupancy level. This saturation is more pronounced for H3K4me3 occupancy compared to PolII, consistent with published results from human CD4+ T-cells [40]. We also concur with the result of that report showing that H3K4me3 is more predictive for lower values of CpG, as the separation of different expression levels is more pronounced for lower CpG quantiles. This lack of separation for higher CpG content suggests that another regulatory input variable is necessary, generally, to predict expression at high levels of H3K4me3.

Similarly, at the highest expression and highest CpG content, we only observe a weak dependence of expression on PolII occupancy, so that another avenue of regulation is required to account for the difference. We find it notable that PolII occupancy varies by a factor of 100 across the observed range of expression (scale in Figure 6g), while H3K4me3 occupancy increases by a factor of 1,000 from low to high expression (scale in Figure 6h). This suggests that the H3K4me3 mark is a more precise predictor of expression than PolII occupancy for lower levels of expression. A clustering analysis (Additional file 6) recapitulates these general trends of expression prediction (compare with Figure six in [18]).

## Conclusions

We have shown that thousands of expressed transcripts in the mouse liver individually exhibit similar bimodal profiles of PolII ChIP-seq enrichment within 200 bp downstream of annotated TSSs. Genome-wide, this bimodal profile has a sharp peak centered within 10 bp of the annotated transcription start sites and a sharper peak +110±20 bp downstream from the first. We have argued that this pattern is not due to divergent transcription nor known promoter motifs. We also find it unlikely that the bimodal pattern could represent alternative initiation sites since alternative TSSs are observed to scatter more randomly around annotated promoter regions [29] (Additional files 4a and 5). Most convincingly, the characteristic bimodal pattern persisted after we filtered out transcripts with nearby alternative initiation sites (Figure 3a).

Based on our analysis and many complementary reports in the literature, the bimodal profile seems to show separate instances of promoter-captured and proximally-paused PolII. A recent review [24] summarized three categories of mechanisms to account for the pausing phenomenon: kinetic, barrier and interaction. A kinetic model balances the rates of early elongation and the recruitment of pausing factors. A barrier mechanism posits the first nucleosome downstream (at roughly +120 bp) of the TSS as a pausing point for the polymerase. An interaction mechanism is supported by results showing that many accessory proteins seem to affect elongation, including NELF, DSIF, Gdown1 and GNAF [41]. The detailed PolII positioning we found is consistent with the barrier and interaction models since the first nucleosome is typically mapped just downstream of the peak we observe [17,42,43] and the profile of the pausing factor NELF has been shown to be centered near +110 bp [42] (Figure 3). Moreover, a kinetic model of pausing could include abortive initiation events, thought to occur 12 bp after initiation [24], which could be subsumed in the width of the TSS-centered peak we observe for the mammalian genome.

Another study relevant to the barrier mechanism [7] showed that the *Drosophila* permanganate peak 50 bp downstream from the TSS is roughly 50 bp upstream from the PolII peak (Figure five in [18]), which is slightly upstream from the first downstream nucleosome at roughly +135 bp. This is consistent with our putative pausing peak at + 110 bp, though the ChIP-chip implemented in that study was not resolved well enough to see a bimodal pattern matching the one we are reporting. Again, the lower-resolution data are consistent with our result and the HeLa cell result (Figure 3) [23], which are consistent with the barrier model and the interaction model, since the +1 nucleosome is within 10 to 20 bp downstream of the peak at +110 bp and pausing factors co-localize with PolII at the same location [24]. Our higher resolution ChIP-seq data tighten the constraint on the location of the paused polymerase and indicate a separated initiation peak.

While the details of these various assays must be reconciled by further experiments, the unambiguous promoter-proximal occupancy of polymerase we observe is validated by, in any case, many observations that promoter-proximal pausing is a widespread phenomenon in expressed genes [3,26]. Our analysis refines reported PolII localizations in eukaryotes [10,14,18]. In fact, the normalized occupancy difference *ρ* could quantify pausing relative to initiation, similar to the retention score [44] or the pausing index, which is defined as the promoter to body ratio for PolII [5,11,12,16,43,45]. Quantification of our proposed *ρ* (promoter-escape index) and *π* (pause-release index) could also refine modeling of the rate constants for kinetic models of PolII transcription initiation [22].

It remains to be seen whether these promoter-escape and pause-release indices can be validated with more targeted experiments, perhaps with other antibodies for PolII phosphorylation states [46,47] associated with promoter escape. To this end, we re-examined ChIP-seq data from Ser5P and 8WG16 antibodies from another PolII ChIP-seq study [47] using our methods. We found that the genomic coverage and thus the resolution of the PolII-Ser5P profile is, again, too low for determining whether the phosphorylated PolII agrees with the location of our putative pausing peak. Our study highlights the importance of improving the resolution of ChIP-seq occupancy profiles for PolII and for associated factors relevant for transcription regulation. As sequencing experiments become more precise, we expect that the generally rich, reproducible structure of these profiles will advance quantitative understanding of the transcription checkpoints between recruitment and elongation.

## Materials and methods

### PolII ChIP-seq

We used existing PolII ChIP-seq libraries provided by the CycliX consortium [22], originally prepared using chromatin extracted from C57/BL6 wild-type mouse livers from five biological replicates. Immunoprecipitation was achieved with an antibody against the N-terminal domain RPB2 subunit (sc-67318 from Santa Cruz Biotechnology, Dallas, Texas, USA). The improvement in resolution and coverage seen here comes from utilization of an Illumina HiSeq 2000 machine, instead of the previous-generation Genome Analyzer II (GAII), which increased the total number of PolII reads from 40 million to 200 million. H3K4me3 ChIP libraries were not resequenced on the HiSeq 2000 machine. Mapping was performed with Bowtie [48], allowing three mismatches and at most five hits on the genome. Quantification of these reads was done with the considerations used for Additional file 2 and described here. After mapping the reads onto the mouse genome, we shifted tag positions [49] accounting for insert sizes, length of the sequencing reads and relative orientation of the reads with respect to TSS (Additional file 2). We emphasize that the reads must be properly aligned to resolve the bimodal genome-wide profile in polymerase localization near the TSS (also discussed in [14]). Multiple reads per position were retained here, as high-coverage sequencing saturates the PolII signal at promoters, notably for highly expressed (determined by mRNA microarray probes) transcripts (Additional file 7a,b). In fact, the proportion of genes with a completely saturated signal between 80 and 120 bp downstream of the TSS increased significantly with the new, high-coverage sequencing (Additional file 7c,d). The improved resolution in the bimodal pattern was not significantly biased due to GC content (Additional file 8). Quality control of the samples was performed and validated with Bioanalyzer checks and FastQC software [50].

### Filtering of raw ChIP-seq signals

A technique for eliminating ChIP-seq artifacts due to PCR amplification is the following. For each genomic position with more than ten tags, we looked for a position within ±50 bp of the tagged position with more than two-thirds of that number of tags. If such a clustering of high tag counts is not found, the signal at the highly tagged position is ignored. This method removes most artifacts without sacrificing the sequencing depth provided by the HiSeq machine. Since the effect of this filter was negligible, we did not apply it to the analysis here, and we take this as an indication that this quantification is not significantly affected by PCR amplification of tags.

### Reanalysis of HiSeq paired-end ChIP-seq datasets in HeLa cells from Liu *et al*. [23]

Using paired-end reads [23] we determined the center position of a subset of fragments between 150 and 170 bp in size and made a binned histogram of these center positions at each genomic position. A selection was made for transcripts with non-zero RNA-seq signal [51] while transcripts with a nearby PolII signal were removed to avoid bidirectional transcription artifacts. These minimal selection criteria do not qualitatively affect the bimodal shape of interest.

### mRNA microarrays

Expression data from mRNA was obtained from the CycliX consortium, as described in [22]. Briefly, mRNA from five biological replicates (C57/BL6 mouse liver) at one time point was pooled and quantified on Mouse Gene 1.0ST arrays from Affymetrix (Santa Clara, California, USA). We found that the expression obtained by the microarray is very highly correlated with the expression inferred from RNA deep sequencing (Additional file 9).

### Bidirectional promoters and nearby transcription start sites

We define bidirectional promoters from the mm9 (NCBI37) annotation as pairs of opposite-strand promoters closer than 1 kb (green in Figure 3). A subset of bidirectional promoters between 170 and 200 bp distant from the TSS in question was also selected (blue in Figure 3). A list of promoters without any other TSSs or PAS within ±1 kb was selected for Figures 2, 4, 5 and 6 (black in Figure 3) and for the models (see Additional file 10).

### Gaussian fit of high-resolution PolII pattern

Based on the average PolII ChIP-seq profile for expressed genes (Figure 1c), we fitted an empirical sum of two Gaussians for each individual gene (Additional file 3). The model is defined with four parameters (optimized for each gene): the distance between the two means of the distributions, the standard deviation of each distribution and the ratio between the maxima of the two distributions. The quality of the fit is the cross-correlation between the model, defined by the computed parameters, and the data. Each parameter is re-estimated until the quality of the fit stabilizes (Additional file 3i).

### RNA deep sequencing

RNA-seq data was generated from an RNA sample (ZT02) provided by Frédéric Gachon (University of Lausanne) from previously published work [52]. The RNA was isolated through hybridization of poly(A) regions on magnetic beads. Isolated RNA was fragmented and cDNA was synthesized with random primers. Sequencing of these non-strand-specific 100-bp paired-end reads was performed with an Illumina HiSeq 2000 machine at the Lausanne Genomic Technologies Facility for two replicates. RNA-seq mapping was performed with the STAR mapping protocol [53] using default options for the mm9 (NCBI37) genome. Quality control of the samples was performed and validated with Bioanalyzer assays and the FastQC read quality analysis package [50]. In particular, the RNA integrity number (RIN) is 8.5 from the Bioanalyzer and 97% of the reads were mapped, with 81% mapping uniquely. Only uniquely mapped reads were used for the bioinformatic analysis.

### Rectangular fit of the transcription start site with RNA-seq

We use a rectangular step function to find the best fit to the RNA-seq coverage subject to the following constraints. The downstream side of the rectangle is fixed for every gene at the first splice junction. Next, a correlation is computed between the rectangular function and the read density, allowing the starting position of the rectangle to vary within ±200 bp of the annotated TSS. The position maximizing the correlation is accepted as the new best-fit TSS. If the correlation reaches a maximum further than 120 bp from the previously annotated TSS, the model fit is considered unacceptable.

### Sequence element identification

The CpG number and the GC content are taken from the FASTA [54] of the mm9 (NCBI37) genome in a window of ±1 kb around TSSs. For the TATA score, see Ref. [22] (Additional file 7).

### Data availability

Raw data for the HiSeq PolII ChIP-seq and RNA-seq are available on the GEO public repository [GEO:GSE58443]. RNA expression data and lower resolution ChIP-seq data for PolII and H3K4me3 [22] are available at [GEO:GSE35790]. Processed CycliX data are also currently available from the database [55].

## Abbreviations

a.u.: arbitrary units; bp: base pair; ChIP-seq: chromatin immunoprecipitation followed by deep sequencing; DSIF: DRB-sensitivity inducing factor; GAII: Genome Analyzer II; kb: kilobase; NELF: Negative elongation factor; PAS: polyadenylation site; PCR: polymerase chain reaction; *p*_*d*_: total number of reads in downstream feature in bimodal peak; *π*: putative pause-release index, the ratio of gene-body PolII density to promoter-proximal downstream peak enrichment density; PIC: pre-initiation complex; PolII: RNA polymerase II; *p*_*u*_: total number of reads in upstream feature in bimodal peak; *ρ*: putative promoter-escape index, the normalized ratio of the bimodal occupancy difference; RNA-seq: RNA isolation followed by deep-sequencing; TSS: transcription start site; Ubxn2b: UBX domain protein 2B.

## Competing interests

The authors declare that they have no competing interests.

## Authors’ contributions

MQ wrote and ran analyses, produced the figures and co-wrote the manuscript. CG analyzed RNA-seq data. FN designed the analyses and conceived the project. KBG directed the analyses and wrote the manuscript. All authors approved the manuscript.

## Supplementary Material

Additional file 1 **Table of selection criteria for genes used in the different figures.** This table provides criteria on expression, isolation or fitting parameters used to select genes for the different figures of the paper. Click here for file

Additional file 2 **Genomic profiles of PolII around TSSs for the four different kind of tags and specific shifts.** The first sign (+ / -) refers to the coding strand of the gene and the second sign to the mapping strand of the tag. **(a)** Average profiles without any shifting. **(b)** Profiles after shifting tags mapping on the minus strand by 100 base pairs (sequencing length). **(c)** Profiles after applying an additional shift of 74 base pairs (computed by correlation, representing half of the mean tag length) to realign the four different profiles. a.u., arbitrary units. Click here for file

Additional file 3 **Examples of bimodal PolII promoter-proximal profiles with different peak height ratios and convergence of the algorithm. (a–h)** ChIP-seq profiles of PolII in a 2-kb window centered on the TSS for eight different mm9 (NCBI37) annotated genes, chosen to have the first peak very near the TSS without correction and with mRNA microarray expression in the top quartile. CpG content (NCBI37) and the location of the 110 bp separation are included. **(a, b, c)** Promoters with peak height ratios 1.64, 1.23, 2.20, **(d, e, f)** peak height ratios 0.87, 0.66, 0.66, and **(g, h)** peak height ratios 1.01, 0.92. **(i)** Convergence of the fitting score for 100 iterations for 20 random genes. a.u., arbitrary units. Click here for file

Additional file 4 **Bidirectional promoters RNA-seq and distribution. (a)** RNA-seq coverage from the first position of each sequenced read near TSSs for expressed transcripts, separated into the following categories: all expressed (red, 10,111 genes, >6.0 microarray units), expressed and isolated with no other TSS or PAS within 1 kb (black, 3,070 genes), those expressed with an oppositely directed TSS less than 1 kb upstream (green, 1,605 genes) and those expressed with an oppositely directed TSS between 170 and 200 bp upstream (blue, 91 genes). The irregular downstream signal is due to individually highly expressed transcripts. **(b)** Histogram of the position of the nearest upstream opposite TSSs for all genes. a.u., arbitrary units. Click here for file

Additional file 5 **Correlation between PolII and CAGE transcription start sites.** Correlation between the peak position of 5^′^ ends of CAGE tags (data from FANTOM liver sample) and the first peak of our PolII ChIP-seq profiles, relative to NCBI37 annotated TSS for expressed genes with a CAGE signal. Transcripts are selected according to expression (>6.0), existence of CAGE signal near their TSSs, and a good PolII fit (>0.8) with our model (*N*=620). Transcripts with alternative TSSs are also excluded. Click here for file

Additional file 6 **Clustering analysis of ChIP-seq signal with sequence and mRNA expression.** This clustering shows general trends and some very specific high-TATA promoters with high expression, in contrast with most TATA promoters showing low expression. PolII seems to be highly correlated with expression, as does H3K4me3, as expected [27]. Click here for file

Additional file 7 **Managing duplicates for high-coverage genomic profiles of PolII around the TSS. (a)** Mean PolII profile for 10,773 genes (coding on plus strand) keeping duplicate tags (black), without duplicates (green), same but scaled by 5 (blue) and with duplicates filtered (see Materials and methods) (red). **(b)** Same as **(a)**, but for the top 10% microarray signal promoters. **(c)** Histogram of mean tag occupation between +70 and +130 (biggest peak region) for the top 10% of expressed promoters for lower coverage data. **(d)** Same as **(c)** but for 5 × higher-coverage sequencing showing saturation of signal. a.u., arbitrary units. Click here for file

Additional file 8 **CpG and GC-content profiles around TSSs for stratification in Figure **6**.** It is known that high GC content can bias Illumina sequencing towards fewer reads. These panels show that the GC content near the promoter did not create the observed bimodal feature. **(a)** Genomic profiles of CpG for classes of promoters separated by quantiles formed on the number of CpG in a ±1-kb window around the TSSs. The TATA >2% group is defined as the genes with the top 2% TATA score. **(b)** Stacked histograms of the number of CpG in a ±1-kb window around the TSSs. A small number of TATA genes are shown in orange. **(c)** Same as **(a)** but for mean GC content. **(d)** Same as **(b)** but for mean GC content. The colors are for the same groups as in **(a)**. A small number of TATA genes are barely visible in the stacked histograms. Click here for file

Additional file 9 **Difference between microarray and RNA-seq to determine expression. (a)** Correlation between microarray and RNA-seq inferred expression (11,741 genes with both measures available). **(b)** Correlation between microarray and RNA-seq inferred expression after quantile normalization of both datasets, *R*^2^=0.87, *P*<2.2×10^-16^. **(c)** Correlation between average PolII signal at TSSs and microarray expression. **(d)** Correlation between average PolII signal at TSSs and RNA-seq expression. **(e)** Average profile of PolII at TSSs for quantiles of expression based on microarray data. **(f)** Average profiles of PolII at TSSs for quantiles of expression based on RNA-seq. a.u., arbitrary units. Click here for file

Additional file 10 **Table of genes and properties selected for analysis.** This table provides transcript name, microarray expression, TATA score, CpG count, GC content and nearest gene-neighbor distances. Some genes are not classified as alone because they have a nearby alternative TSS or PAS of a neighboring gene. Click here for file
