# Supplementary material for: Characteristic bimodal profiles of RNA polymerase II at thousands of active mammalian promoters
Source: Genome Biol. 2014 Jun 12;15(6):R85. doi: 10.1186/gb-2014-15-6-r85 (PMC4197824; doi:10.1186/gb-2014-15-6-r85)
Supplement: Additional file 1 — Table of selection criteria for genes used in the different figures. This table provides criteria on expression, isolation or fitting parameters used to select genes for the different figures of the paper. [file gb-2014-15-6-r85-S1.pdf]

List of transcript populations used in each figure panel with descriptions of filters. The conclusions of the manuscript do not depend in detail on these selections – they are made only to draw distinctions and highlight features.

Figures

1. Separation in three groups according to expression
   High expression (microarray signal > 8) (5144 genes)
   Low expression (microarray signal < 6) (6054 genes)
   Same as a) and b) Middle (signal between 6 and 8) (4967 genes divided in two)

2. a) Isolated and microarray signal>6 (3070)
   b) All with microarray signal>6 (10111)
   Bidirectional with opposite TSS between -1000bp and -1bp microarray signal>6 (1605)
   Bidirectional with opposite TSS between -200bp and -170bp microarray signal>6 (91)
   re-analysis of HeLa cell data from Liu *et al*. JBC 2014
   (c,d) N=4446: selected for non-zero RNA-seq expression and eliminating nearby oppositely directed transcription – the bimodal result does not depend qualitatively on this selection

3. b) Isolated, microarray available and non-null PolII signal within ±1000bp (6475)
   c-f) Same as b) but with quality of fit > 0.85 and model-bounded interpeak distance (1475)

4. a-d) Microarray signal>6, isolated and RNA-seq signal less than 1000 (this removes 280 genes with too high signal) (2790)

5. c) Black: Microarray signal>6, isolated, very high quality of fit > 0.85, both shifts smaller than 120bp in absolute value and interpeak distance between 80 and 160bp (731)
   Red: Same as c) but extremely high quality of fit > 0.98 (183)
   Blue: Same as c) but very high TATA score (12)
   d) Same as c) but with total forward RNA signal less than 1000 (this removes 107 genes with too high signal) (620)

6. Isolated, low expression (microarray signal < 6) (3414)
   Isolated, medium expression (microarray signal between 6 and 8) (1669 genes divided in two groups of same size)
   Isolated, high expression (microarray signal > 8) (1401)

Supplementary figures

Additional File 2 — (a-c) all (N=21518)

Additional File 3 — single genes

Additional File 4 — (a) red, N=10111, > 6.0 microarray units; black, N=3070: isolated; green, N=1605 bidirectional; blue, N=91: nearby bidirectional.
(b) all (N=21518)

Additional File 5 — N=620: expression (> 6.0), CAGE signal near TSSs, and PolII fit score > 0.8); transcripts with alternative TSS are excluded.

Additional File 6 — all (N=21518)

Additional File 7	(a-d) all (N=21518)
Additional File 8	(a-d) All with microarray and RNA-seq data available (N=11741)
Additional File 9	(a-b) TATA (score>-5), expressed (between 6 and 9), RNA-seq signal less than 1000 and isolated (152)<br>c-d) random non-TATA (score<-5), expressed (between 6 and 9), RNA-seq signal less than 1000 and isolated (152)<br>e-f) random non-TATA (score<-5), expressed (between 6 and 9), RNA-seq signal less than 1000 and isolated (152)
