# Supplementary figures and images for: Characteristic bimodal profiles of RNA polymerase II at thousands of active mammalian promoters
Source: Genome Biol. 2014 Jun 12;15(6):R85. doi: 10.1186/gb-2014-15-6-r85 (PMC4197824; doi:10.1186/gb-2014-15-6-r85)

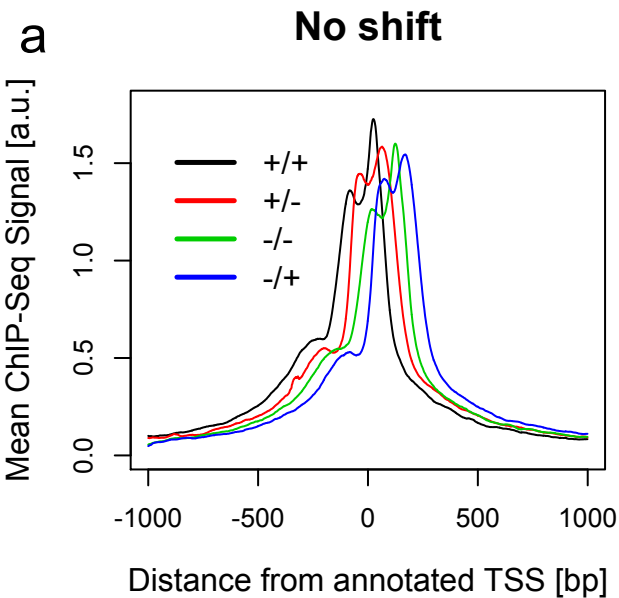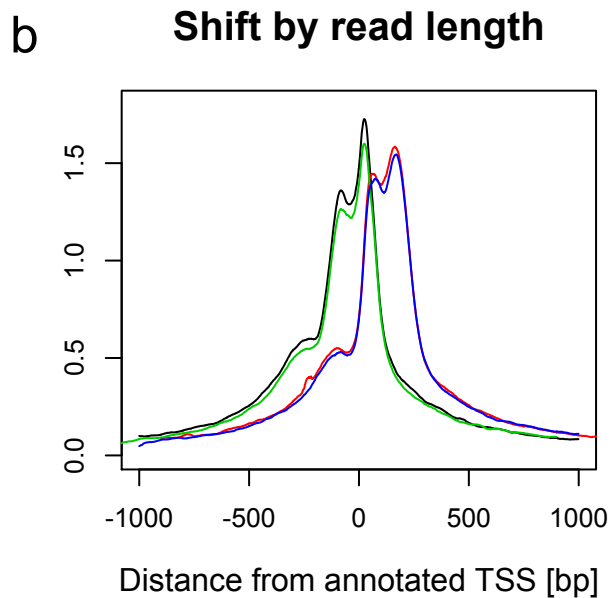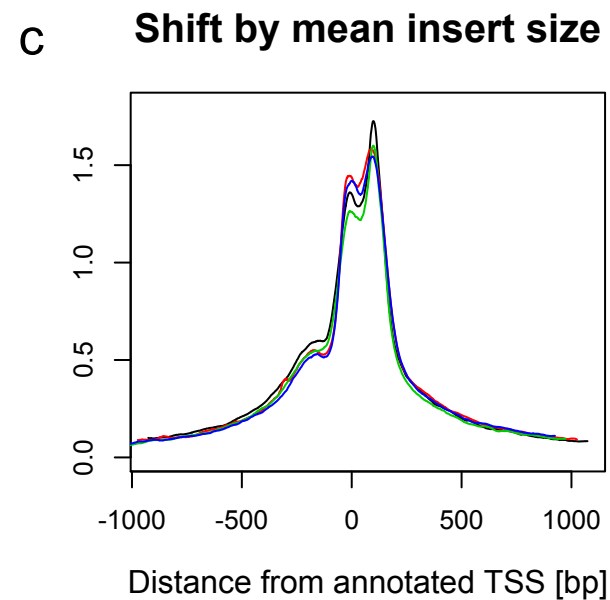

Supplement: Additional file 2 — Genomic profiles of PolII around TSSs for the four different kind of tags and specific shifts. The first sign (+ / -) refers to the coding strand of the gene and the second sign to the mapping strand of the tag. (a) Average profiles without any shifting. (b) Profiles after shifting tags mapping on the minus strand by 100 base pairs (sequencing length). (c) Profiles after applying an additional shift of 74 base pairs (computed by correlation, representing half of the mean tag length) to realign the four different profiles. a.u., arbitrary units. [file gb-2014-15-6-r85-S2.pdf]

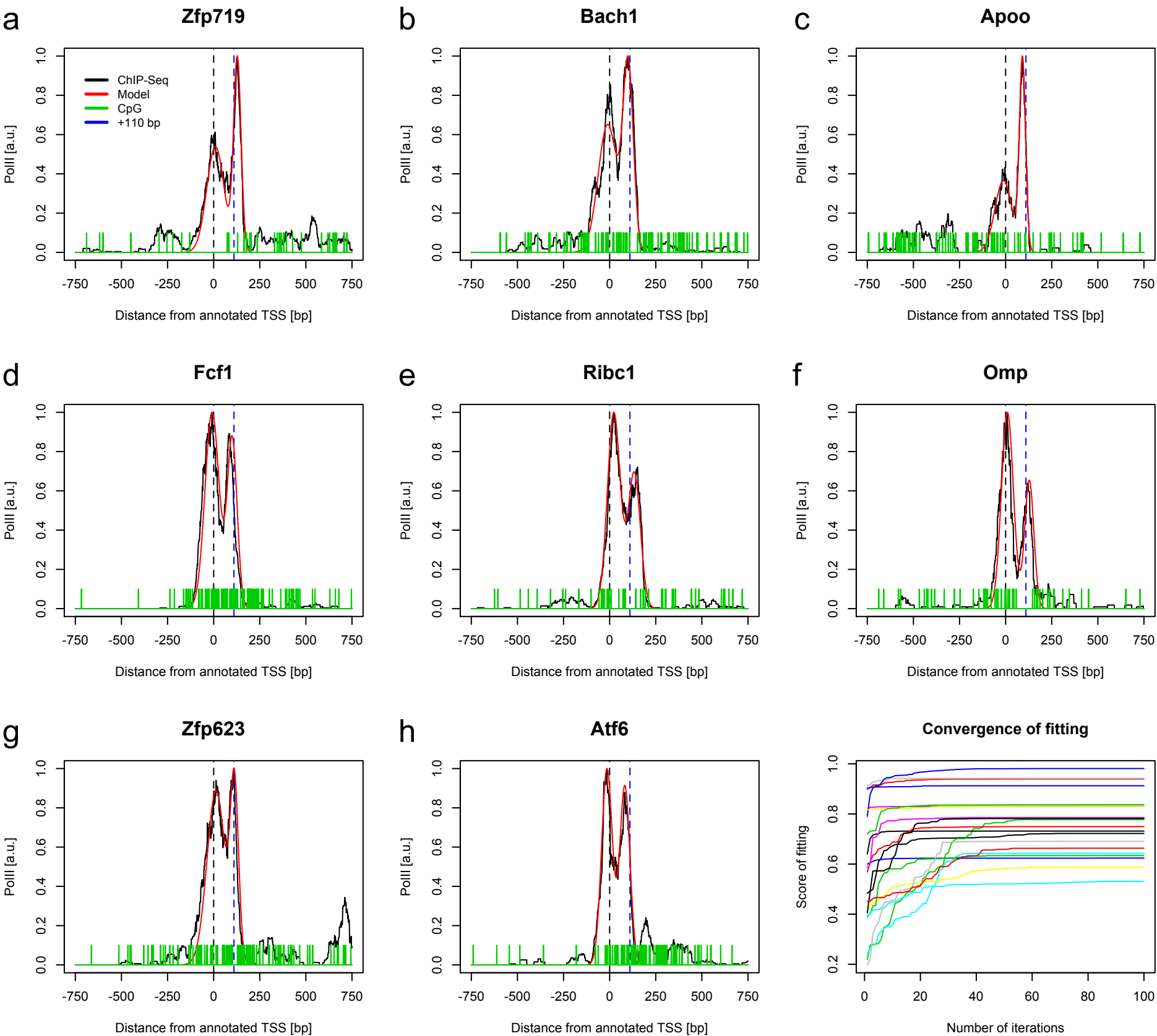

Supplement: Additional file 3 — Examples of bimodal PolII promoter-proximal profiles with different peak height ratios and convergence of the algorithm. (a–h) ChIP-seq profiles of PolII in a 2-kb window centered on the TSS for eight different mm9 (NCBI37) annotated genes, chosen to have the first peak very near the TSS without correction and with mRNA microarray expression in the top quartile. CpG content (NCBI37) and the location of the 110 bp separation are included. (a, b, c) Promoters with peak height ratios 1.64, 1.23, 2.20, (d, e, f) peak height ratios 0.87, 0.66, 0.66, and (g, h) peak height ratios 1.01, 0.92. (i) Convergence of the fitting score for 100 iterations for 20 random genes. a.u., arbitrary units. [file gb-2014-15-6-r85-S3.pdf]

**a****RNA**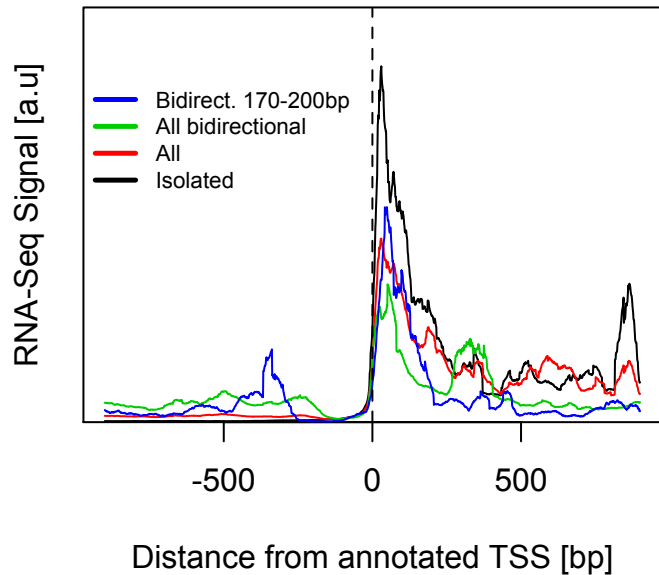**b****Bidirectional promoters**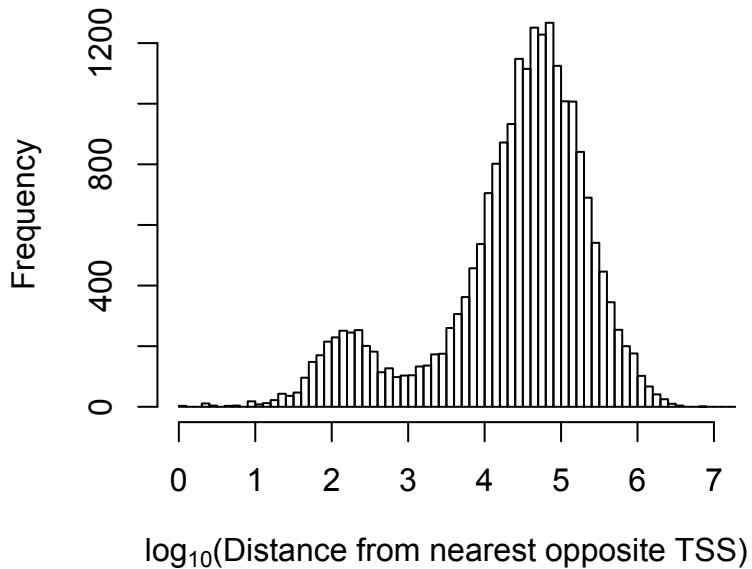

Supplement: Additional file 4 — Bidirectional promoters RNA-seq and distribution. (a) RNA-seq coverage from the first position of each sequenced read near TSSs for expressed transcripts, separated into the following categories: all expressed (red, 10,111 genes, >6.0 microarray units), expressed and isolated with no other TSS or PAS within 1 kb (black, 3,070 genes), those expressed with an oppositely directed TSS less than 1 kb upstream (green, 1,605 genes) and those expressed with an oppositely directed TSS between 170 and 200 bp upstream (blue, 91 genes). The irregular downstream signal is due to individually highly expressed transcripts. (b) Histogram of the position of the nearest upstream opposite TSSs for all genes. a.u., arbitrary units. [file gb-2014-15-6-r85-S4.pdf]

$R^2=0.43$

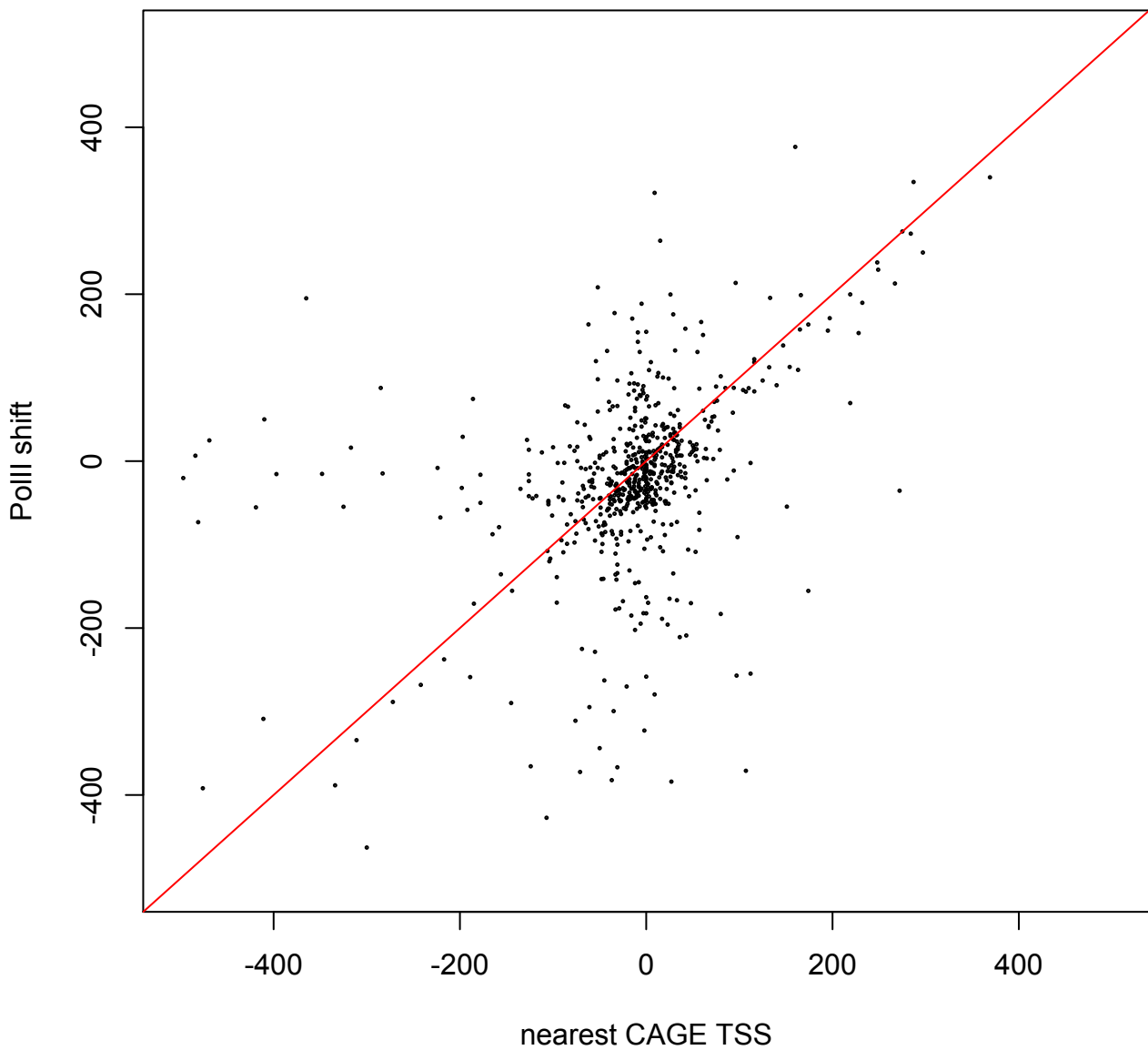

Supplement: Additional file 5 — Correlation between PolII and CAGE transcription start sites. Correlation between the peak position of 5′ ends of CAGE tags (data from FANTOM liver sample) and the first peak of our PolII ChIP-seq profiles, relative to NCBI37 annotated TSS for expressed genes with a CAGE signal. Transcripts are selected according to expression (>6.0), existence of CAGE signal near their TSSs, and a good PolII fit (>0.8) with our model (N=620). Transcripts with alternative TSSs are also excluded. [file gb-2014-15-6-r85-S5.pdf]

**CpG   H3K4   TATA   POL2**

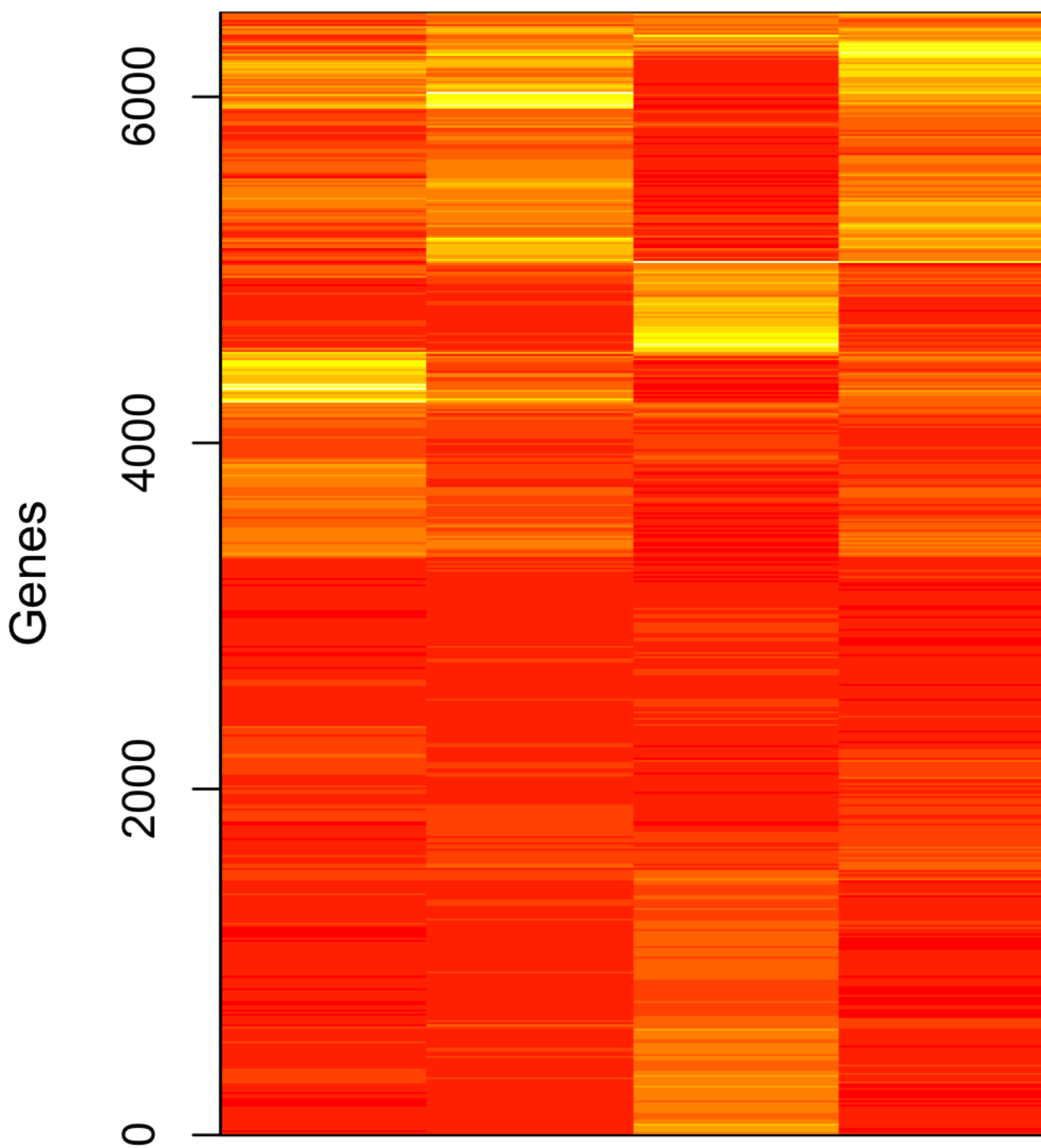

**Expression classified by clustering**

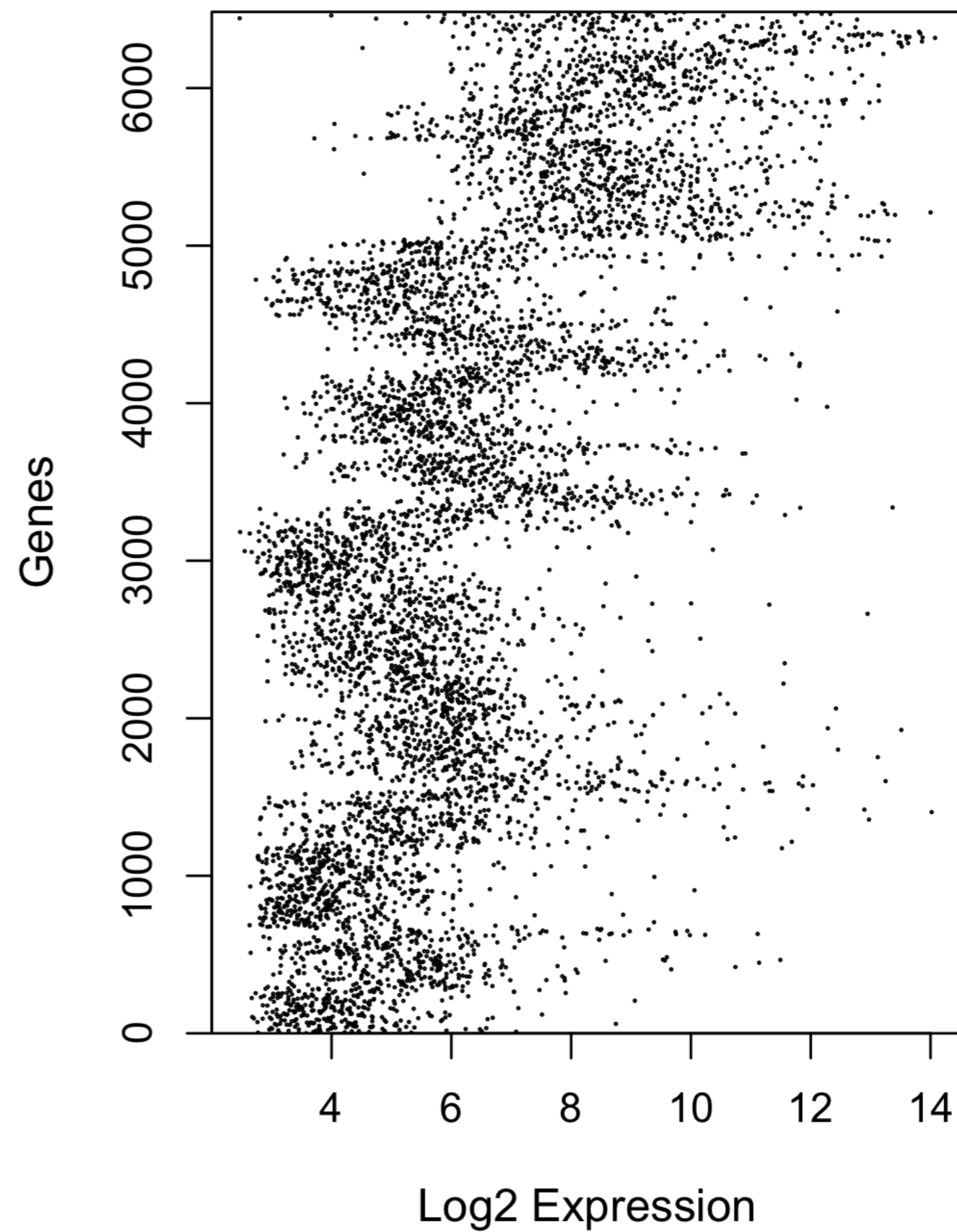

Supplement: Additional file 6 — Clustering analysis of ChIP-seq signal with sequence and mRNA expression. This clustering shows general trends and some very specific high-TATA promoters with high expression, in contrast with most TATA promoters showing low expression. PolII seems to be highly correlated with expression, as does H3K4me3, as expected [27]. [file gb-2014-15-6-r85-S6.pdf]

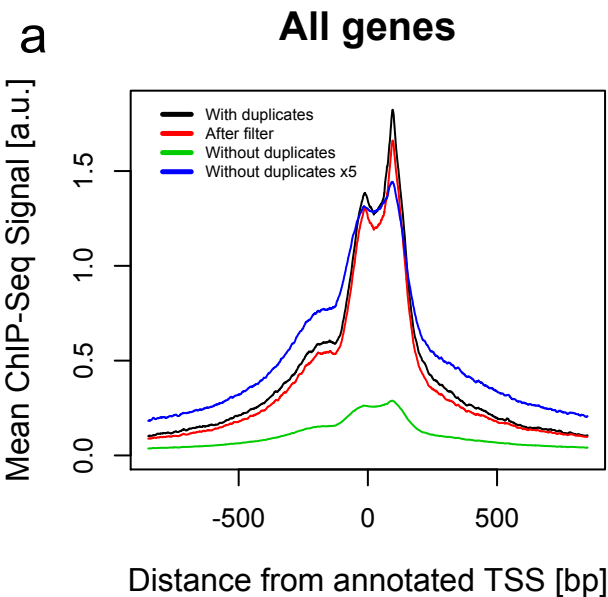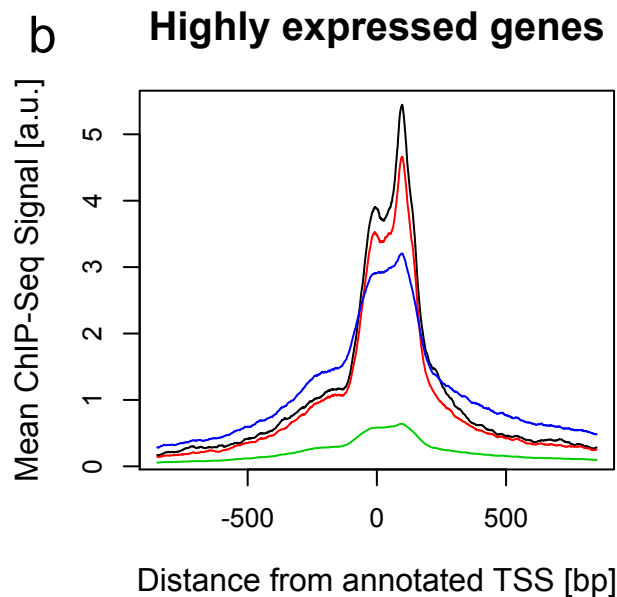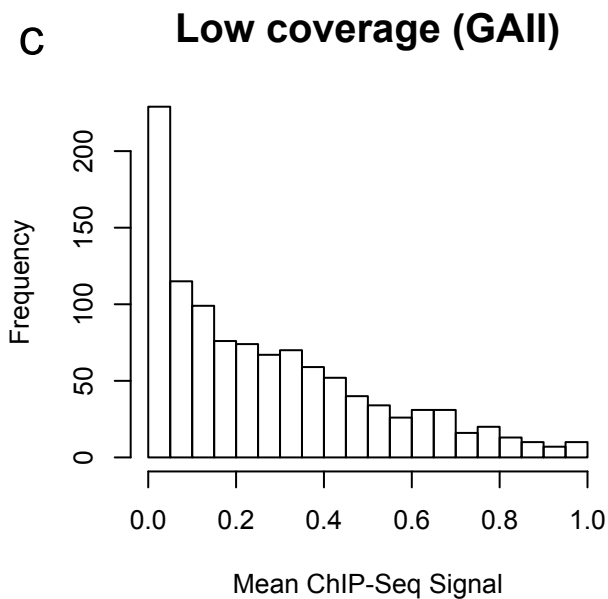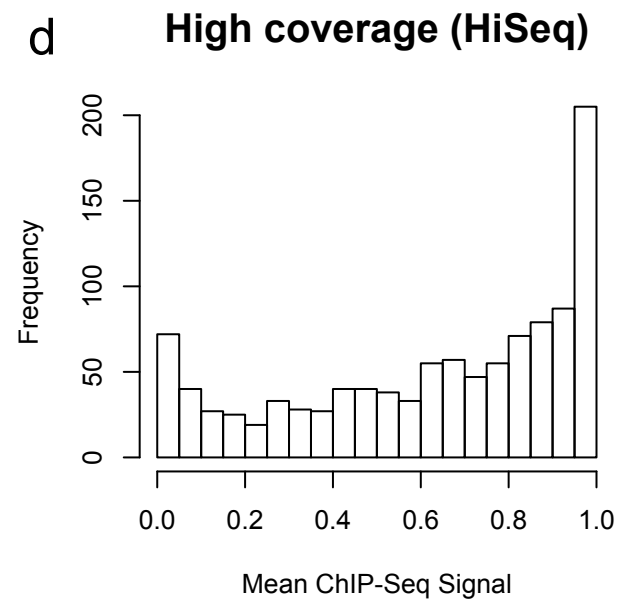

Supplement: Additional file 7 — Managing duplicates for high-coverage genomic profiles of PolII around the TSS. (a) Mean PolII profile for 10,773 genes (coding on plus strand) keeping duplicate tags (black), without duplicates (green), same but scaled by 5 (blue) and with duplicates filtered (see Materials and methods) (red). (b) Same as (a), but for the top 10% microarray signal promoters. (c) Histogram of mean tag occupation between +70 and +130 (biggest peak region) for the top 10% of expressed promoters for lower coverage data. (d) Same as (c) but for 5 × higher-coverage sequencing showing saturation of signal. a.u., arbitrary units. [file gb-2014-15-6-r85-S7.pdf]

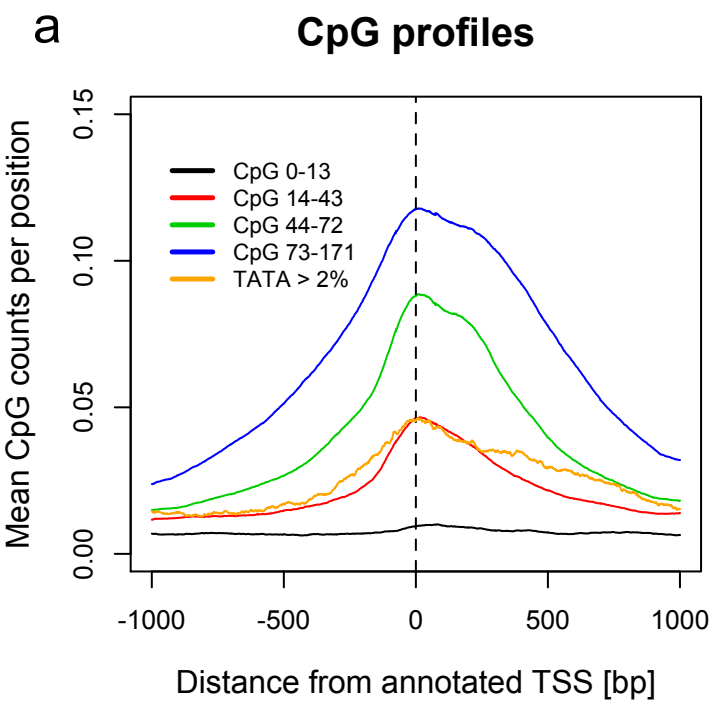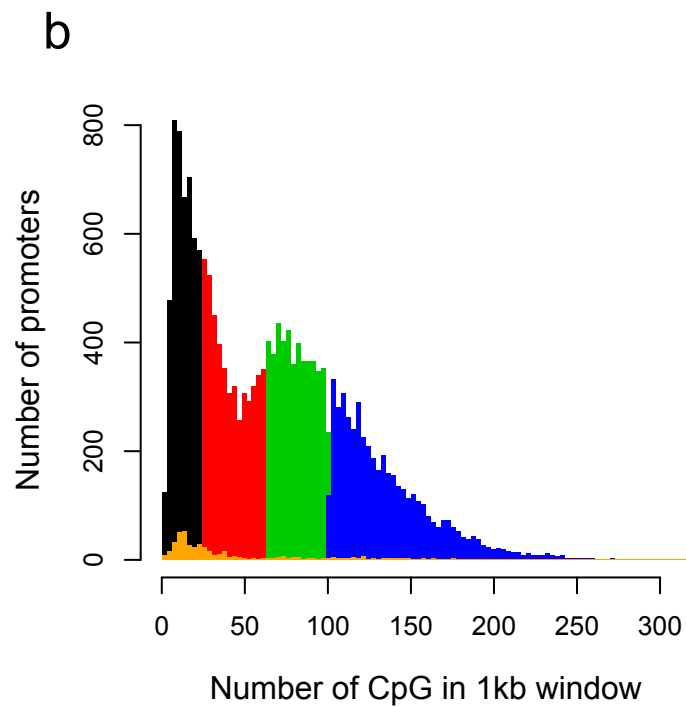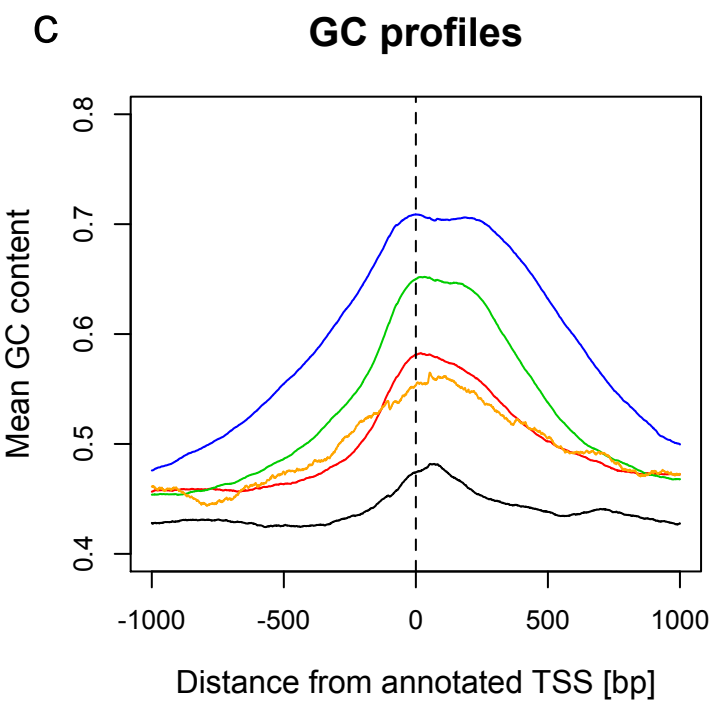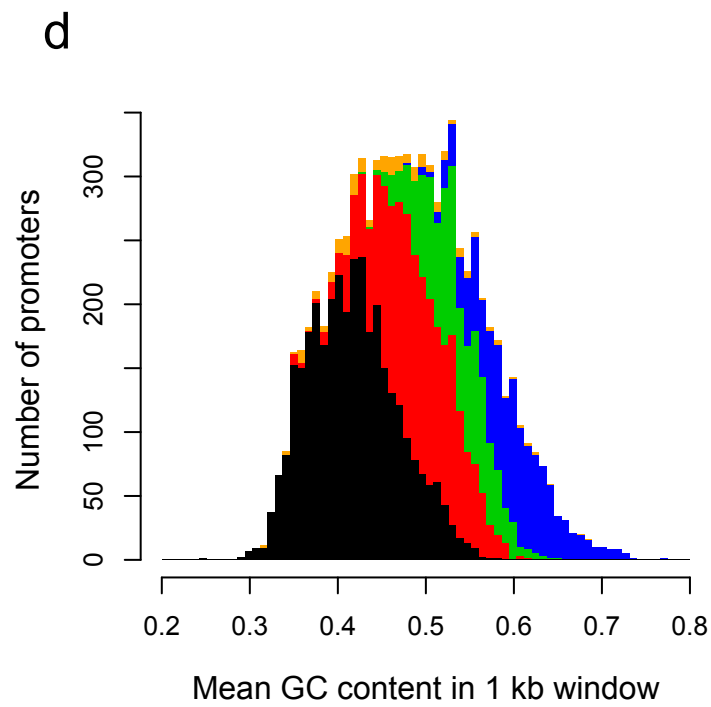

Supplement: Additional file 8 — CpG and GC-content profiles around TSSs for stratification in Figure 6. It is known that high GC content can bias Illumina sequencing towards fewer reads. These panels show that the GC content near the promoter did not create the observed bimodal feature. (a) Genomic profiles of CpG for classes of promoters separated by quantiles formed on the number of CpG in a ±1-kb window around the TSSs. The TATA >2% group is defined as the genes with the top 2% TATA score. (b) Stacked histograms of the number of CpG in a ±1-kb window around the TSSs. A small number of TATA genes are shown in orange. (c) Same as (a) but for mean GC content. (d) Same as (b) but for mean GC content. The colors are for the same groups as in (a). A small number of TATA genes are barely visible in the stacked histograms. [file gb-2014-15-6-r85-S8.pdf]
