# Supplementary material for: Characteristic bimodal profiles of RNA polymerase II at thousands of active mammalian promoters
Source: Genome Biol. 2014 Jun 12;15(6):R85. doi: 10.1186/gb-2014-15-6-r85 (PMC4197824; doi:10.1186/gb-2014-15-6-r85)
Supplement: Additional file 9 — Difference between microarray and RNA-seq to determine expression. (a) Correlation between microarray and RNA-seq inferred expression (11,741 genes with both measures available). (b) Correlation between microarray and RNA-seq inferred expression after quantile normalization of both datasets, R2=0.87, P<2.2×10^-16^. (c) Correlation between average PolII signal at TSSs and microarray expression. (d) Correlation between average PolII signal at TSSs and RNA-seq expression. (e) Average profile of PolII at TSSs for quantiles of expression based on microarray data. (f) Average profiles of PolII at TSSs for quantiles of expression based on RNA-seq. a.u., arbitrary units. [file gb-2014-15-6-r85-S9.pdf]

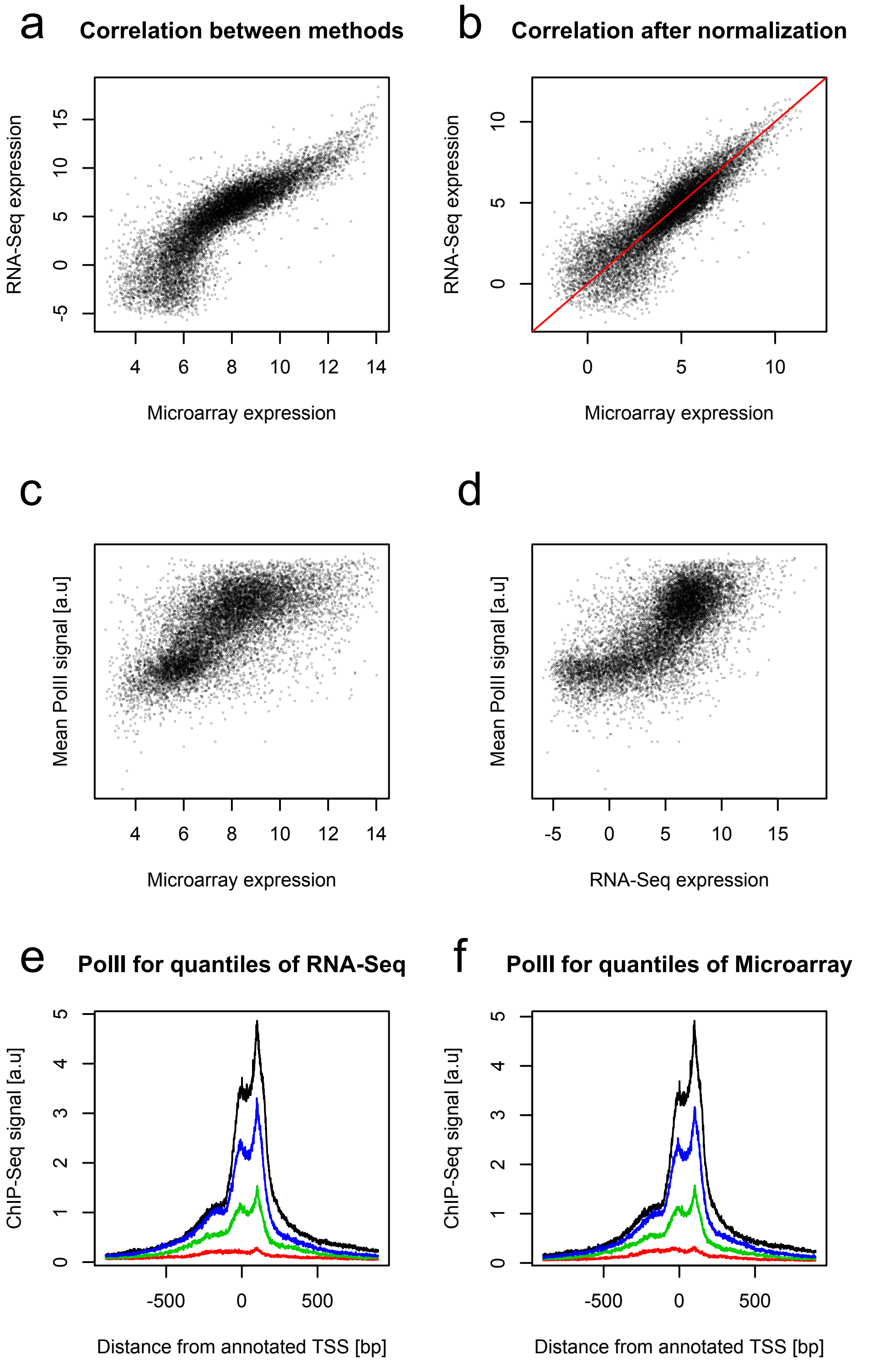
a
Correlation between methods
RNA-Seq expression
Microarray expression
b
Correlation after normalization
RNA-Seq expression
Microarray expression
c
Mean PolII signal [a.u]
Microarray expression
d
Mean PolII signal [a.u]
RNA-Seq expression
e
PolII for quantiles of RNA-Seq
ChIP-Seq signal [a.u]
Distance from annotated TSS [bp]
f
PolII for quantiles of Microarray
ChIP-Seq signal [a.u]
Distance from annotated TSS [bp]
